# BST2/Tetherin Enhances Entry of Human Cytomegalovirus

**DOI:** 10.1371/journal.ppat.1002332

**Published:** 2011-11-03

**Authors:** Kasinath Viswanathan, M. Shane Smith, Daniel Malouli, Mandana Mansouri, Jay A. Nelson, Klaus Früh

**Affiliations:** Vaccine and Gene Therapy Institute, Oregon Health and Science University, Beaverton, Oregon, United States of America; University of Alabama at Birmingham, United States of America

## Abstract

Interferon-induced BST2/Tetherin prevents budding of vpu-deficient HIV-1 by tethering mature viral particles to the plasma membrane. BST2 also inhibits release of other enveloped viruses including Ebola virus and Kaposi's sarcoma associated herpesvirus (KSHV), indicating that BST2 is a broadly acting antiviral host protein. Unexpectedly however, recovery of human cytomegalovirus (HCMV) from supernatants of BST2-expressing human fibroblasts was increased rather than decreased. Furthermore, BST2 seemed to enhance viral entry into cells since more virion proteins were released into BST2-expressing cells and subsequent viral gene expression was elevated. A significant increase in viral entry was also observed upon induction of endogenous BST2 during differentiation of the pro-monocytic cell line THP-1. Moreover, treatment of primary human monocytes with siRNA to BST2 reduced HCMV infection, suggesting that BST2 facilitates entry of HCMV into cells expressing high levels of BST2 either constitutively or in response to exogenous stimuli. Since BST2 is present in HCMV particles we propose that HCMV entry is enhanced via a reverse-tethering mechanism with BST2 in the viral envelope interacting with BST2 in the target cell membrane. Our data suggest that HCMV not only counteracts the well-established function of BST2 as inhibitor of viral egress but also employs this anti-viral protein to gain entry into BST2-expressing hematopoietic cells, a process that might play a role in hematogenous dissemination of HCMV.

## Author Summary

Human Cytomegalovirus (HCMV) persistently infects a large proportion of the human population without causing any symptoms. The establishment and maintenance of HCMV in infected individuals is thought to be facilitated by the ability of HCMV to modulate innate and adaptive immune responses by the host. BST2, aka Tetherin, was recently shown to be an innate immune response molecule that is induced by the antiviral cytokine interferon. BST2 has been shown to prevent the release of many different viruses, including the human immunodeficiency virus and Ebola virus, from infected cells by tethering the viral envelope to the host cell membrane. Unexpectedly however, we observed that BST2 had the opposite effect on infection by HCMV. Cells expressing BST2 became more susceptible to infection with HCMV. Thus, HCMV seems to use this antiviral protein to gain access to cells that naturally express high levels of BST2 such as macrophages.

## Introduction

Human cytomegalovirus (HCMV), a β-herpesvirus, maintains a lifelong, asymptomatic infection in immunocompetent hosts but is an opportunistic pathogen in immunocompromised individuals [1], [2]. HCMV is also the leading infectious cause of congenital birth defects in neonates [3]. Moreover, in post-transplant patients HCMV is capable of causing disseminated disease in most organs and tissue types [4], [5], [6]. Thus, HCMV is able to infect a wide range of host cells. However, the host factors required for viral entry into different cell types are incompletely understood. Initially the virus attaches to heparan sulphate proteoglycans, followed by virion surface glycoproteins interacting with their cellular receptors that include integrins and the EGF receptor along with other as yet undefined molecules in cholesterol rich membrane micro-domains [7]. The two known pathways of HCMV entry are fusion with the plasma membrane and endocytosis. The respective pathway used is dependent on the cell type and viral glycoprotein composition [8], [9]. The role of cellular receptors in each of these processes is largely unknown, and it is likely that yet to be identified cellular proteins will be involved in viral entry processes.

BST2 (Bone marrow stromal cell antigen 2) was initially thought to be involved in normal and malignant B cell differentiation since this protein is expressed on bone marrow stromal cells and multiple myeloma cells [10]. However, the murine homologue was later shown to be highly expressed by plasmacytoid dendritic cells suggesting a role in innate immunity. Moreover, it was shown that BST2 is an IFN inducible protein that can act as a ligand to ILT7, a receptor on dendritic cells that modulates IFN production [11], [12]. The first indication that BST2 might be involved in the host defense against viruses was implied by our finding that BST2 was downregulated by the immune evasion molecule K5/MIR2, a transmembrane E3 ubiquitin ligase of Kaposi's sarcoma associated herpesvirus (KSHV) that targets multiple host cell immunoreceptors for destruction via ubiquitination [13], [14]. Subsequently, it was demonstrated that BST2 represented the interferon-induced host cell factor responsible for preventing release of HIV-1 lacking Vpu, [15], [16], [17]. Prior to this work, Vpu was known to eliminate CD4 and MHC-I via ubiquitin-mediated processes [18]. Based on this finding, many unrelated enveloped viruses were recently shown to be restricted by BST2, including the α-retrovirus RSV, the β-retroviruses MPMV and HERV-K, the δ-retrovirus HTLV-1, the spumaretrovirus PFV, the filoviruses Marburg and Ebola, the arenavirus Lassa, non-human primate retro viruses, and the endogenous β-retroviruses of sheep enJSRV [19], [20], [21], [22]. In addition to K5 and Vpu, several BST2-antagonists were discovered in other viruses, including HIV-2 Env, simian immunodeficiency virus (SIV_mac/smm_) Nef, SIV_tan_ Env, and Ebola GP [17], [19], [21], [23], [24].

BST2 is a heavily glycosylated, type II transmembrane protein. It has a short cytoplasmic N-terminal region, a transmembrane region, a coiled coil extracellular domain and a C-terminal glycosylphosphatidylinisotol (GPI) anchor [25]. This topology of BST2 with a transmembrane domain and a GPI anchor is rather unusual and is shared by only one other protein, an isoform of the prion protein [25], [26]. BST2 forms intermolecular disulfide bridges with conserved extracellular cysteines in the coiled-coil domain [27]. It was further suggested that BST2 forms a picket fence-like structure having its transmembrane domain located on the periphery and the GPI anchor inside the lipid raft [25]. This flexible structure of BST2 is thought to be important for preventing virions from budding by retaining the transmembrane domain in the cell membrane and the GPI anchor incorporated into the virion during the budding process [28].

Here, we examine whether BST2 restricts HCMV release. We report the surprising observation that increased titers of HCMV are obtained from supernatants of BST2-expressing fibroblasts, a finding that is in stark contrast to observations reported to date for all other viruses implying that HCMV efficiently overcomes any anti-viral function of BST2. On closer inspection, we found that increased viral release was a downstream effect of BST2 enhancing viral entry and thereby HCMV gene expression and replication. A potential role for BST2-mediated HCMV entry in viral pathogenesis and dissemination is suggested by the finding that BST2 enhances entry of HCMV into monocytes that constitutively express high levels of BST2. Our data thus suggest that HCMV uses this IFN-induced anti-viral protein to increase infection of monocytic cells, which play a central role in HCMV latency, reactivation and dissemination.

## Results

### BST2 enhances virus infection

**Figure 1:**
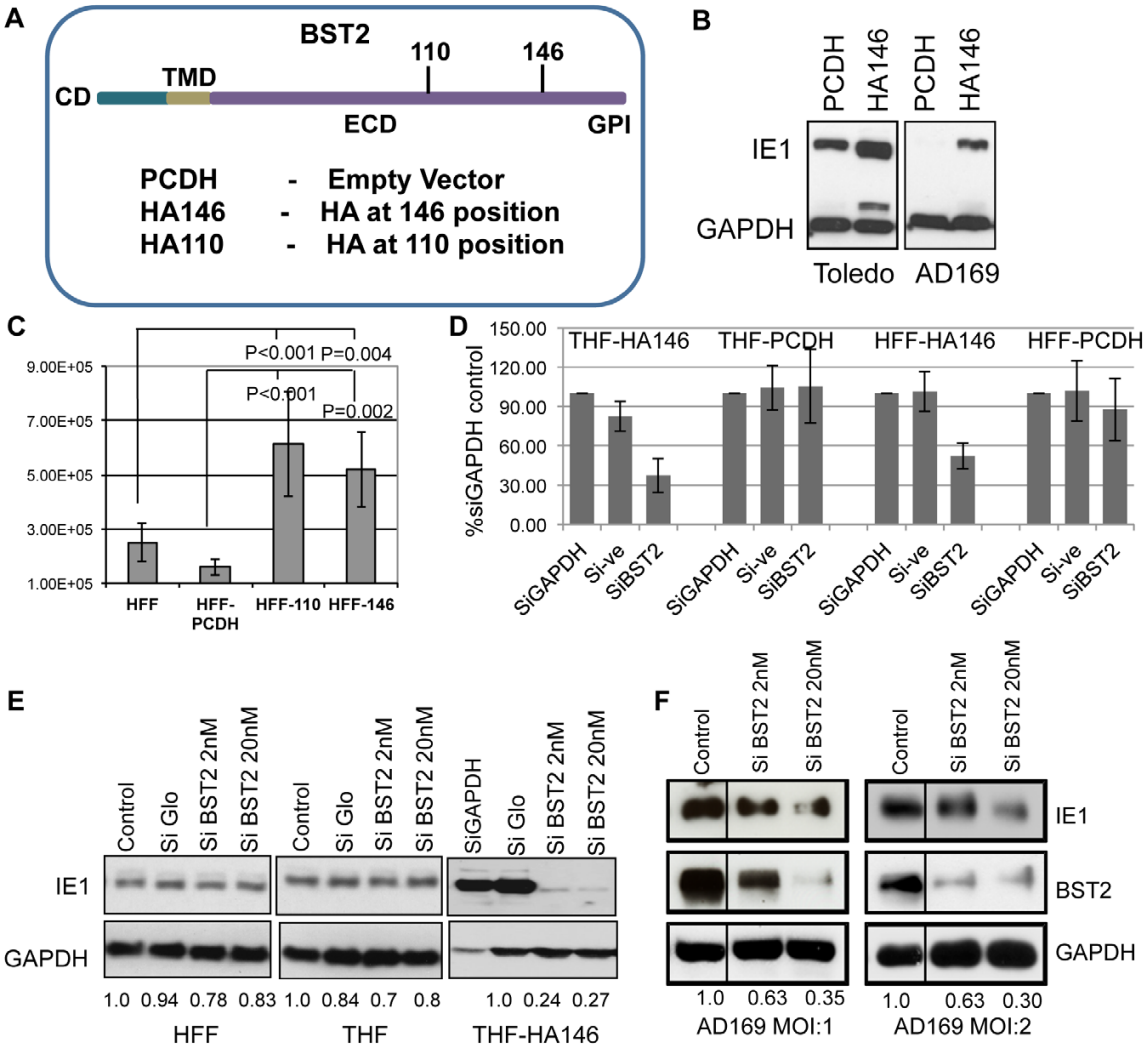
BST2/Tetherin enhances HCMV infection and release. A) A schematic representation of the BST2 constructs used in this study. B) Immunoblot for HCMV-IE1 or GAPDH of HFFs exposed to supernatants of HCMV-infected THFs expressing BST2-HA146 or vector control. Supernatants were obtained after infection with HCMV strains AD169 or Toledo (MOI = 3) for 72 hrs. C) Viral titers in supernatants of AD169-infected HFFs stably expressing the indicated BST2 constructs. Virus was titrated by end point serial dilution assay. D) Percentage of GFP positive cells of THF-HA146, THF-PCDH, HFF-HA146 and HFF-PCDH cells transfected with the indicated siRNAs 72 hours prior to infection with AD169-GFP for 24 h. GFP levels in GAPDH controls were set to 100%. E) Immunoblot for IE1 and GAPDH in HFFs, THFs and THF-HA146 cells transfected with the indicated siRNA 72 h prior to infection with HCMV Toledo for 8 h. F) Immunoblot for IE1, BST2 and GAPDH in HFF-HA146 cells transfected with indicated siRNAs prior to infection with HCMV AD169 at the indicated MOI for 8 h. For control 40 nM silencer negative control (Ambion) was used. The numbers below the blots in E) and F) show the relative IE1 band intensity compared to GAPDH (using ImageJ) normalized to control siRNA.

To examine whether the ability of BST2/Tetherin to prevent release of a wide spectrum of enveloped viruses would extend to HCMV, we examined the supernatant of HCMV-infected telomerized human foreskin fibroblast (THFs) cells stably expressing an HA-tagged version of BST2 (THF-HA146) (Fig. 1A). Two different strains of HCMV were used: the lab-adapted strain AD169 and the clinical isolate Toledo. BST2-expressing THFs or control THFs (THF-PCDH) were infected with an MOI of 3, and the supernatants were harvested at 72 hours post-infection (hpi). To determine the presence of infectious virus, we added the supernatant to fresh primary human foreskin fibroblasts (HFFs). After 8 h, cells were washed, trypsinized and viral infection was monitored by analysis of viral immediate early gene (IE1) expression in immunoblot. Unexpectedly, we observed higher IE1 levels in HFFs exposed to supernatants from BST2-expressing THF-HA146 cells compared to the supernatant obtained from control THF-PCDH cells (Fig. 1B). This indicated that there was an increased amount of infectious virus particles released in the presence of BST2, which is contrary to the observation of restricted virus release of other enveloped viruses. To confirm this observation, we determined the amount of infectious virus released into the supernatant by plaque assay. We infected two different HFF-lines expressing BST2 with HA tags at AA positions 110 or 146 (Fig. 1 A). Each of the BST2-expressing HFF-lines, as well as a control-transfected HFF line, was infected with AD169 at an MOI of 3. The supernatants were collected 72hpi and the concentration of HCMV plaque forming units (PFU) was determined by dilution on HFFs. Compared to the control-transfected HFFs, approximately five times more PFU were recovered from each of the BST2 expressing cells (Fig. 1C). Thus, infectious virus released in the supernatant increased in the presence of BST2 irrespective of the position of the HA tag.

To determine whether increased presence of virus in the supernatant was due to increased infection of the BST2-expressing fibroblasts or increased infectivity of released virus we knocked down BST2 in THF-HA146 and HFF-HA146 cells using a previously described siRNA [14] prior to infection with AD169-GFP (MOI = 1). The number of infected cells was monitored by flow-cytometry for GFP. At 24 hpi the cells were harvested and the number of GFP-positive cells was determined. The results showed that inhibiting BST2 expression reduced the number of GFP-positive cells whereas knockdown of the cellular protein GAPDH or control siRNA had no effect (Fig. 1D). Furthermore, BST2 siRNA did not affect the number of GFP-positive cells in control fibroblasts. Thus, BST2 enhanced an early event in the viral life cycle rather than promoting egress or infectivity of released virus.

Since these data suggested that early or immediate early events were modulated by BST2 we compared the expression of the viral immediate early gene 1 (IE1) in BST2-expressing THFs to control THFs or HFFs by immunoblot. For control of host cell gene expression and as loading control we included immunoblots for GAPDH. Furthermore, we treated each cell line with control siRNA (siGLO) or with siRNA to BST2 prior to infection with the HCMV strain Toledo. As shown in Fig. 1E, there was a significant increase of IE1 protein recovered from BST2-expressing THFs compared to both HFF and THF. In contrast, targeted knockdown of BST2, but not of GAPDH or control siRNA treated cells, reduced the levels of IE1 expression in the BST2-transfectants but not in the control cells (Fig. 1E, F). Taken together these data demonstrate that BST2 expression increased viral infection of fibroblasts and that this increase of infection was responsible for increased virus production of these cells.

### BST2 enhances virus entry

**Figure 2:**
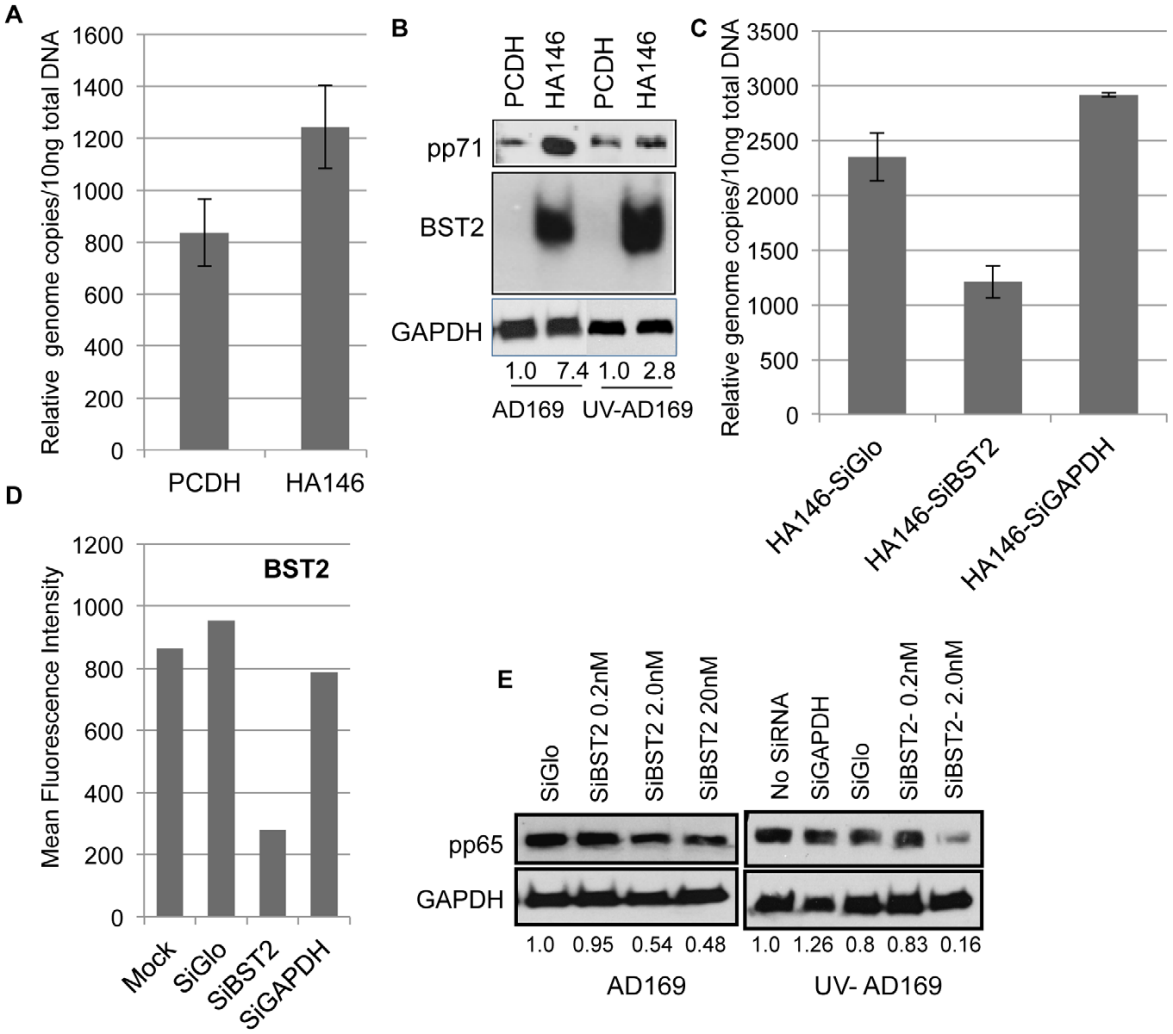
BST2/Tetherin enhances HCMV entry into fibroblasts. A) Relative viral genome copies were quantified using Taqman probes in the BST2-expressing or control THFs exposed to HCMV AD169 (MOI = 2) at 37°C for 90 min followed by a citric acid (pH = 3) wash. B) Immunoblot for pp71, BST2 and GAPDH of BST2-expressing or control THFs exposed to live or UV-inactivated HCMV-AD169 (MOI = 2) at 37°C for 90 min followed by a citric acid (pH = 3) wash. Band intensities of pp71 were compared to GAPDH. C) Relative viral genome copies were measured in BST2 expressing THF-HA146 cells treated with the indicated siRNAs for 72 h prior to infection with HCMV for 90 min. Infection conditions were as in A). D) In parallel cultures, mean fluorescent intensity levels of surface expression of BST2 was determined by flow cytometry for siRNA treated HA146-BST2 transfected THFs. E) In a similar experiment as in C) the cells were treated with indicated siRNAs prior to infection with live or UV-inactivated HCMV AD169 (MOI-2). The level of tegument protein pp65 was analyzed by immunoblot compared to GAPDH.

Since BST2 is a protein that recycles between the cell surface and intracellular compartments [25] it was conceivable that enhanced infection of BST2-expressing fibroblasts was caused by BST2 enhancing viral entry. Upon fusion of herpes virions with plasma or endosomal membranes, proteins of the viral tegument, a protein-rich compartment layered between the capsid and the envelope, are released into the cytoplasm together with the viral capsid [29]. Therefore, we determined the amount of the major tegument proteins pp71 and pp65 recovered from BST2-expressing or control THFs immediately after virus infection and prior to onset of immediate early gene expression. Additionally, we monitored the amount of viral genomes present in cells by qPCR. THF-PCDH and THF-HA146 cells were infected with AD169 (MOI = 3) for 2 h at 37°C, and then the cells were washed with citric acid buffer (pH = 3) to remove adhered virions from the cell surface. Cells were trypsinized, lysed and analyzed for genomic DNA by qPCR (Fig. 2A) or the tegument protein pp71 by immunoblot (Fig. 2B). At 2hpi, BST2-expressing cells contained an increased amount of viral genome copies as well as tegument protein pp71 (Fig. 2A, 2B). In contrast, when BST2 expression was decreased by siRNA in THF-HA146 (Fig. 2D), reduced amounts of genomic DNA (Fig. 2C) and tegument protein pp65 (Fig. 2E) was recovered. These results are consistent with viral entry being increased in the presence of BST2. To rule out that increased tegument proteins were due to increased viral gene expression we exposed cells to UV-inactivated AD169 which is still able to attach to and enter cells but unable to express viral genes. Similar to infection with untreated virus, increased amounts of pp71 were recovered from THF-HA146 infected with UV-inactivated virus as compared to THF-PCDH (Fig. 2B), whereas BST2 knockdown decreased recovery of pp65 from THF-HA146 infected with UV-inactivated HCMV (Fig. 2E).

### Modulation of IFN-dependent and IFN-independent BST2 induction by HCMV

**Figure 3:**
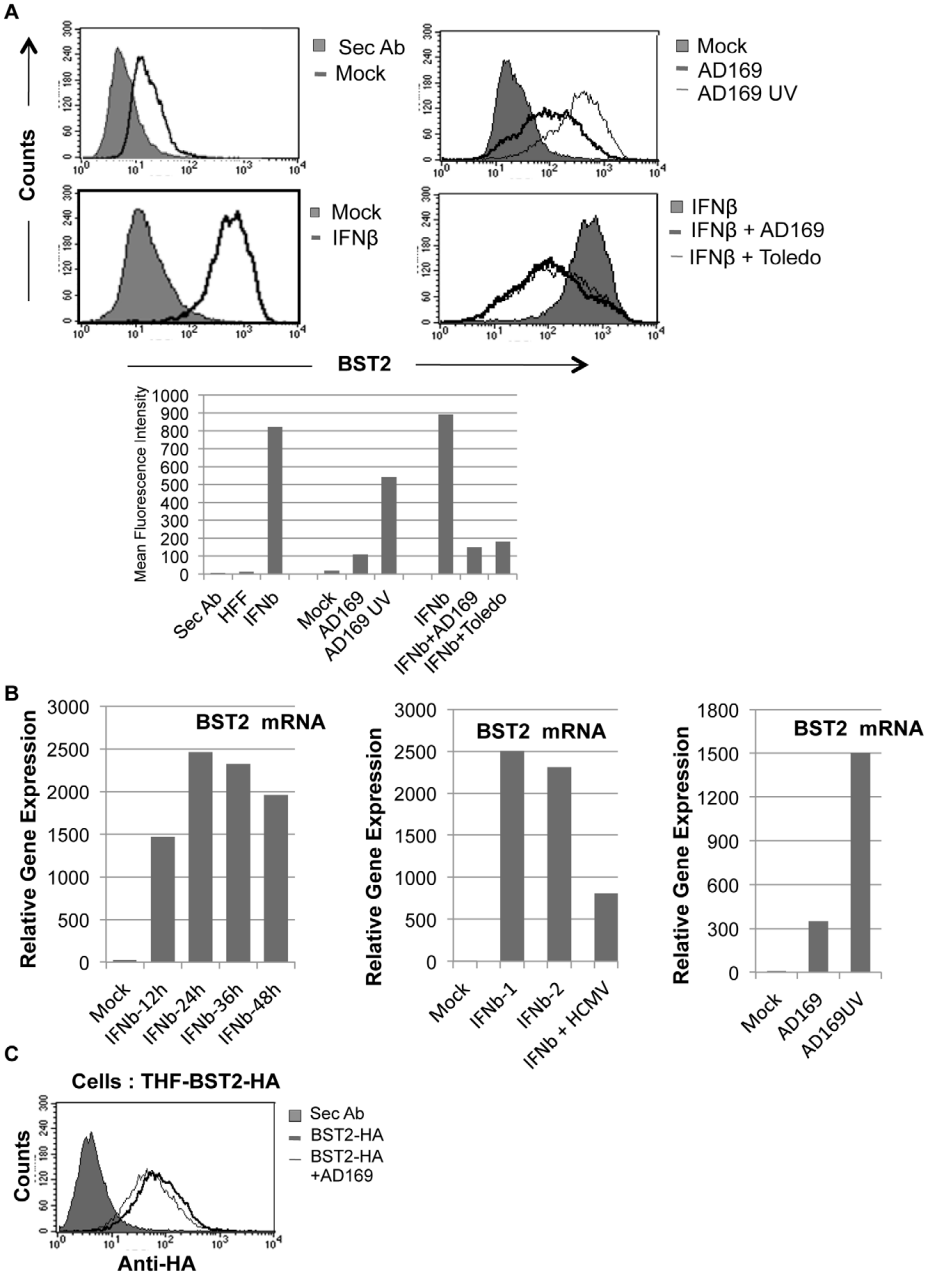
HCMV induces BST2 independent of IFN, but inhibits IFN-dependent induction. A) Expression of BST2 monitored by flow cytometry using anti-BST2 (HM1.24) antibody is shown in all panels except for the shaded graph in the top left panel that shows staining of secondary antibody alone. Lower panels: Uninfected or HCMV-infected HFFs treated with 500 U of IFNβ for 24 hrs. Right Panels: HFFs infected with indicated viruses (MOI = 3) for 24 hrs. The levels of BST2 are graphically represented below. B) Left panel: qPCR of BST2 mRNA in HFFs treated with 500 U of IFNβ over indicated times. Middle panel: qPCR of BST2 mRNA in HFFs treated with 500 U of IFNβ (two independent experiments) for 24 hours or simultaneously infected with HCMV-AD169 (MOI = 3) for the same amount of time. Right panel: qPCR of BST2 upon infection of HFFs with live or UV-inactivated AD169. C) Flow cytometry of BST2-HA146-expressing THFs uninfected or infected with HCMV-AD169 using anti-HA antibody.

**Figure 4:**
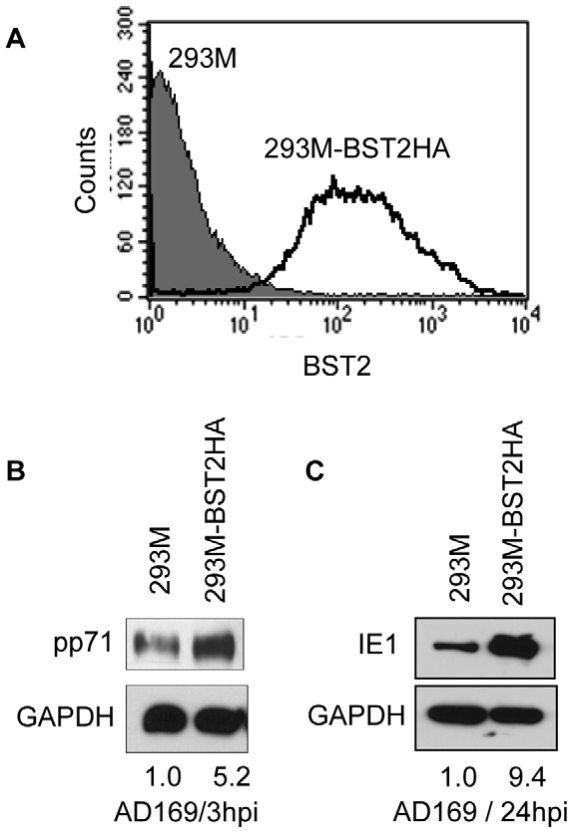
BST2 increases HCMV infection in 293 M cells. A) Surface levels of BST2-HA146 in stably transfected 293 M cells were monitored by flow cytometry with anti-HA. B) Immunoblot of untransfected or BST2HA-transfected 293 M cells infected with AD169 (MOI = 3) and probed for the expression of the tegument protein pp71 or GAPDH at 3hpi after citrate buffer wash. C) Immunoblot for IE1 protein at 24hpi. Relative band intensities are shown to the right of the blots.

HCMV induces interferon (IFN) and IFN-stimulated genes (ISGs), but it is also known to interfere with IFN-dependent, JAK/STAT-mediated ISG-induction [30], [31]. Since BST2 is induced by IFN we were wondering whether HCMV would modulate IFN-independent and IFN-dependent BST2 gene induction during infection. Non-transfected HFFs express low but detectable amounts of BST2 constitutively and BST2 levels can be strongly induced by IFN, as expected (Fig. 3A). Interestingly, induction of BST2 was also observed upon HCMV-infection. Moreover, UV-inactivated HCMV induced BST2 to a much higher level than live HCMV. However, when HFFs were simultaneously treated with IFN and infected with HCMV, HCMV suppressed BST2 expression (Fig. 3A). These results are consistent with the previously reported IRF3-dependent induction of IFN and ISGs by both live and UV-inactivated HCMV [30] whereas IFN-dependent, JAK/STAT-mediated ISG induction is inhibited by live but not UV-inactivated HCMV [32]. This conclusion was further supported when BST2mRNA expression levels were measured by qPCR. Live HCMV induced BST2 mRNA to a lesser degree than UV-inactivated HCMV (Fig. 4B) and BST2 mRNA levels were strongly increased upon IFN treatment. Infection with HCMV reduced this induction (Fig. 4B). Thus, although HCMV interferes with IFN-dependent BST2 gene induction, most likely due to its known interference with JAK/STAT signaling, HCMV induces BST2 mRNA and does not seem to interfere with BST2 protein expression at a post-transcriptional level, in marked contrast to HIV-1 and KSHV. This conclusion is also supported by the fact that HCMV does not downregulate transfected BST2 (Fig. 3C). Thus, BST2 either does not affect HCMV egress, or HCMV counteracts BST2 in a way that does not affect BST2 protein levels or surface expression.

### BST2 enhances HCMV entry into non-permissive 293 cells

To determine whether the entry-enhancing effect of BST2 is limited to fibroblasts, we examined HCMV entry into BST2-expressing HEK293M cells. Unlike HFFs, these cells are not permissive for viral replication and therefore could delineate the role of BST2 in the early events of viral entry versus the later events of viral replication. We generated stable 293 M cells expressing BST2 (Fig. 4A) and infected these or control cells with HCMV-AD169. Viral entry and infection was monitored by immunoblot for pp71 and IE1 as described above. As shown in Fig. 4, compared to 293 M-PCDH control, 293 M-BST2HA cells contained increased amounts of pp71 at 3hpi and IE expression at 24hpi. These results demonstrate that BST2 enhances viral entry independently of cell type and might even contribute to cell tropism.

### BST2 enhances HCMV infection in activated monocytes

**Figure 5:**
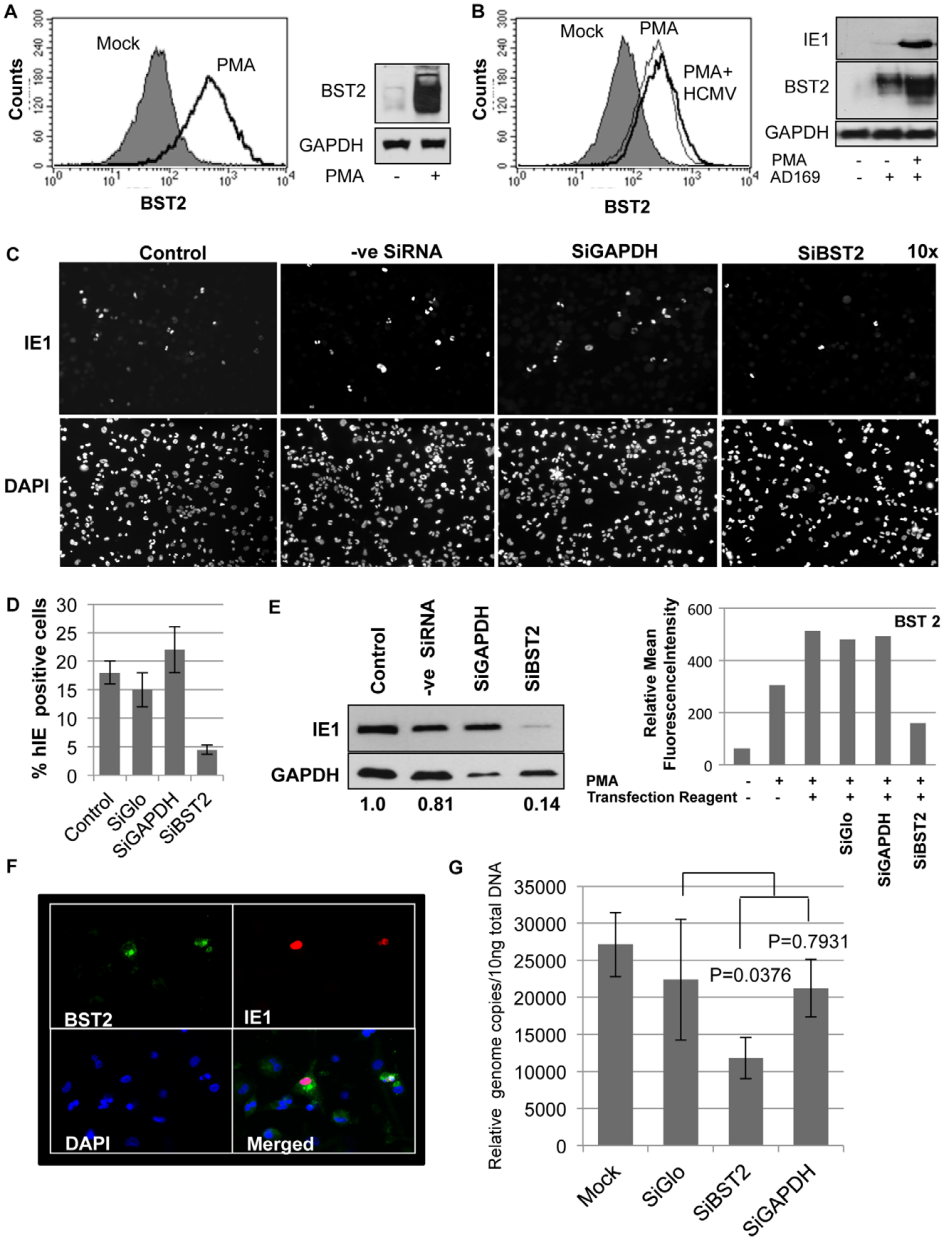
Infection of THP-1 cells with HCMV is BST2-dependent. A) Surface levels of endogenous BST2 upon treatment of THP-1 cells with PMA for 24 hours (left panel) and immunoblot for total BST2 present upon PMA induction (right panel) . B) FACS and immunoblot analysis of THP-1 cells treated with PMA and upon infection with HCMV-AD169. C) IFA of IE1 in PMA-differentiated THP-1 cells treated with indicated siRNAs and infected with HCMV-AD169 for 24 h. Representative fields are shown as 10× magnified frames. D) IE1 positive-cells and DAPI-positive cells were counted from 5 fields using Image J (http://rsbweb.nih.gov/ij/). The average ratio of infected versus total number of cells is shown. E) IE1-immunoblot analysis of a similar experiment as in C. GAPDH-normalized band intensities are given below the bands. The efficiency of BST2 knock down is represented as mean fluorescence intensity of BST2 on the cell surface by flow cytometry analysis. F) IFA of HCMV-infected THP-1 cells treated with low amounts of BST2-siRNA resulting in residual cells expressing BST2. At 24 hpi the cells were probed for BST2, IE1 and DAPI. G) Relative genome copies of HCMV TR at 90 min post-infection of PMA activated THP-1 cells treated with indicated SiRNAs for 72 h. To remove extracellular virus, cells were washed with citric acid buffer (pH = 3) prior to harvesting.

Most somatic cells express low levels of BST2 unless exposed to IFN. However, BST2 is upregulated during the differentiation of cells in the hematopoietic lineage leading to constitutive high level of expression in B cells, T cells, NK cells, and monocyte/macrophages [10], [11]. Particularly pronounced is the expression of BST2 on plasmacytoid dendritic cells which are major IFN producers [11]. Among these cell types, monocytes are thought to be particularly important for HCMV latency, reactivation and dissemination in vivo [33]–[34]. Therefore, we explored whether endogenous BST2 expressed by monocytes would facilitate entry of HCMV into this cell type. In vitro, HCMV is known to infect differentiated monocytes preferably over monocyte precursors [35]. This preference can be recapitulated in the pro-monocytic cell line THP-1 which can be differentiated into a monocytic cell type by treatment with phorbol meristate acetate (PMA), and PMA-induced differentiation correlates with increased infection by HCMV [36]. Interestingly, surface levels of BST2 drastically increased upon PMA treatment of THP-1 cells (Fig. 5A, B). It was previously shown that PMA-treatment of THP-1 cells increased entry of the lab-strain AD169 [36] although only clinical isolates are able to replicate productively in this cell type [37]. Indeed, we observed increased IE1 expression in PMA treated THP-1 cells compared to untreated cells upon infection with AD169 (Fig. 5B). AD169-infection of THP-1 cells induced endogenous BST-2 (Fig. 5B) consistent with the increase of BST-2 observed in AD169-infected fibroblasts (Fig. 3). These data indicated a correlation between PMA-dependent AD169 infection of THP-1 cells and BST2 levels. To determine whether there was a causative relationship between AD169 entry and BST2 levels we knocked down BST2 mRNA levels with siRNA prior to PMA treatment and HCMV infection. THP-1 cells transfected with control siRNAs or BST2 siRNA were treated with PMA and entry of AD169 was monitored for IE1 expression by immunoblot and IFA. As shown in Fig. 5C, IE1 expression was strongly reduced upon BST2 siRNA treatment, whereas reduction of GAPDH or transfection of control siRNA did not have a significant effect. Interestingly, residual IE1 expression in BST2 siRNA-treated cells correlated with residual BST2 expression presumably due to incomplete knockdown in some cells (Fig. 5F). To determine whether BST2 siRNA also reduced entry of endothelial cell and macrophage-tropic clinical isolates, we infected PMA-induced THP-1 cells with the clinical strain HCMV-TR. Compared to control siRNA treated cells, reduced amounts of viral genome copies were recovered from THP-1 cells treated with BST2-specific siRNAs (Fig. 5G). Taken together these data suggest that induction of endogenous BST2 during monocyte differentiation facilitates HCMV entry.

**Figure 6:**
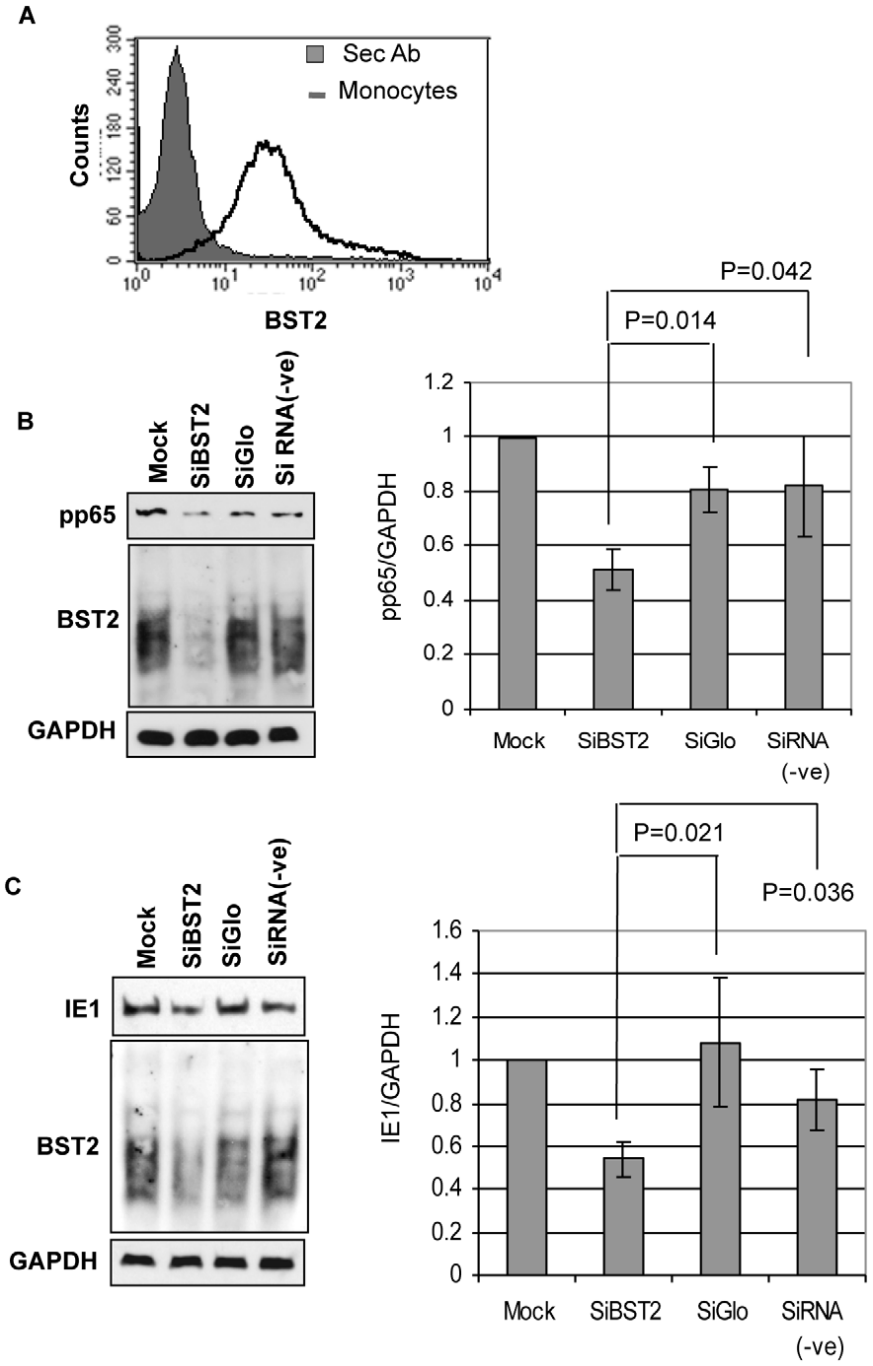
BST2 facilitates HCMV entry into primary human monocytes. A) BST2 expression on adherent monocytes isolated from PBMCs was analyzed by flow cytometry. B) Immunoblot of pp65, BST2 or GAPDH upon treatment of human monocytes with the indicated siRNAs. C) IE1, BST2 or GAPDH expression monitored by immunoblot of human monocytes transfected with the indicated siRNAs. Relative intensities (measured using ImageJ) normalized to GAPDH, from immunoblots of monocytes derived from five different donors are shown to the right in B) and C). Error bars indicate Mean ± SD. A p value of <0.05 was considered significant.

Significant surface levels of BST2 were also observed on primary human monocytes isolated from peripheral blood mononuclear cells (PBMC) (Fig. 6A). To determine whether BST2 facilitated HCMV entry into such primary cells, we knocked down BST2 by transfection of BST2-specific siRNA into monocytes and monitored pp65 (Fig. 6B) or IE1 expression (Fig. 6C) upon infection with HCMV-strain Toledo. Successful knockdown of BST2 was verified by immunoblot (Fig. 6B, C). The experiments were repeated with monocytes from five different donors. We observed a reduced recovery of pp65 (Fig. 6B) and reduced expression of IE1 (Fig. 6C) in BST2-siRNA treated cells, but not in control siRNA treated cells. Therefore, we conclude that BST2 enhances viral entry into primary human monocytes.

### BST2 is present in HCMV virions

**Figure 7:**
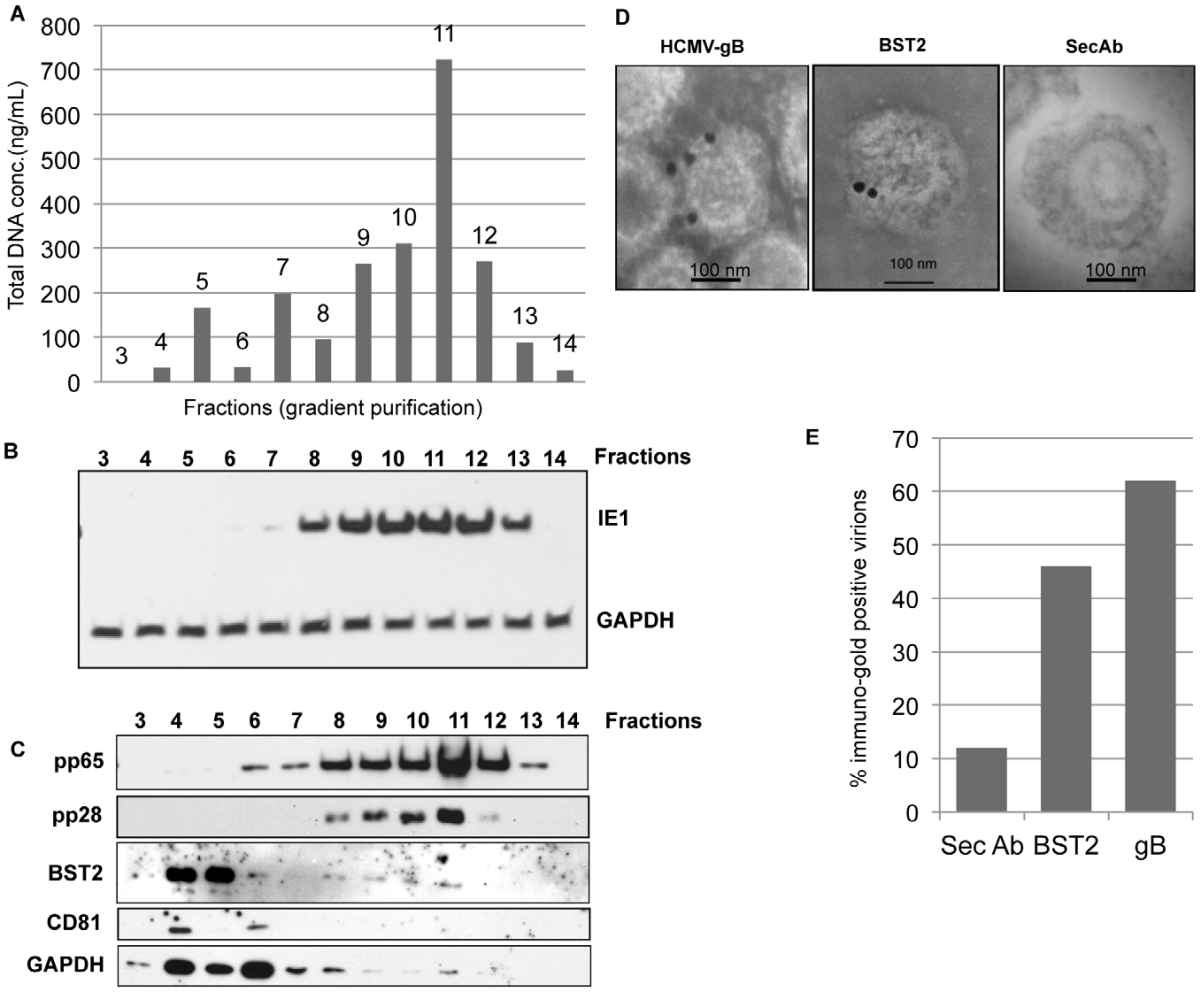
BST2 is present in HCMV virions. A) HCMV preparations were separated by a discontinuous (5–50%) Nycodenz gradient and equal-sized fractions were isolated prior to spectrophotometric analysis of total DNA. B) Immunoblot for IE1 or GAPDH of HFFs infected with each of the fractions. C) Fractions were probed for the viral proteins gB, pp65 and the cellular proteins BST2 (PNGaseF treated), CD81 and GAPDH by immunoblot. D) Immunoelectron microscopy analysis of purified virions for BST2 and HCMV gB. 20 nm gold conjugated secondary antibody was used to detect BST2 and gB. E) Percentage of immunogold positive virions counted from multiple plates for each treatment.

Taken together, above experiments suggest that increased BST2 levels enhance HCMV entry and that this facilitated entry might play an important role in HCMV infection of monocytes. What might be the molecular mechanism by which BST2 enhances entry of viral particles into cells? Given our current knowledge of BST2 as an anti-viral protein that tethers virions to cell membranes, it is conceivable that BST2 increases HCMV entry by a similar tethering mechanism except that here the tether is used to capture HCMV rather than to prevent its release. In the case of HIV-1 it is thought that BST2 needs to be present in both the plasma membrane and the virion and that BST2 prevents budding either by bridging the virion with the membrane or by BST2 forming homo-dimers or –multimers between virion-associated BST2 and BST2 in the host cell membrane. Thus, a prerequisite for the formation of a tether between HCMV and the plasma membrane would be the presence of BST2 in HCMV virions. To determine whether BST2 was present in HCMV particles we fractionated virion preparations via a Nycodenz gradient to separate contaminating cellular membrane fragments and defective viral particles from intact, infectious virions. We tested the concentration of DNA in each of the fractions and detected representative viral and cellular proteins by immunoblot. The DNA concentration was measured with 260/280 nm absorption values and the highest concentration was measured in fractions 9–12 peaking in fraction 11 (Fig. 7A). This correlated with the presence of infectious virus in fractions 8–13 as detected by immunoblot of IE-1 in fibroblasts inoculated with each fraction (Fig. 7B). The same fractions, particularly fraction 11, also contained the highest concentration of the virion proteins pp65 and pp28. In contrast, the cellular transmembrane protein CD81 was only found in lower fractions expected to contain contaminating membranes. To detect BST2 the sample was de-glycosylated using PNGaseF and the blot was probed with rabbit polyclonal anti-BST2. The result showed that BST2 was present in early fractions and reappeared in the fractions 9–11 that contained infectious virus (Fig. 7C). Another cellular protein, GAPDH that was identified by a proteomics study to be present in the virion [38], showed a similar pattern of distribution like BST2 in the gradient, where it was detected in early fractions and reappeared in the later fractions containing live virus. To independently determine the presence of BST2 in the virion membrane we analyzed virion preparations by immune electron microscopy with BST2-specific antibodies using gold-conjugated secondary antibodies. The virus preparation was not permeabilized so that antibodies would only recognize proteins at the surface of the viral envelope. For control, we used the viral envelope protein gB. As shown in Fig. 7D and E, both gB and BST2 were detected on virions. These observations demonstrate that BST2 is present in the virus particle.

## Discussion

In this study we addressed the question whether the β-herpesvirus HCMV is affected by the innate immune response protein bone marrow stromal cell antigen 2 (BST2/HM1.24/CD317/Tetherin), a protein that has been shown previously to restrict the release of a number of unrelated enveloped viruses [19]. Contrary to our expectations, we observed that BST2 is not only incapable of restricting HCMV from egress but it is, in fact, utilized by the virus to gain entry into BST2-expressing cells. Importantly, BST2 facilitated entry of HCMV into hematopoietic cells which are important in the hematogenous dissemination of the virus.

In several previously reported instances, the effect of BST2 can only be observed when viral counter mechanisms are inactivated, e.g. due to genetic deletion of Vpu from HIV-1 or by siRNA treatment against K5 of KSHV [14], [17]. Thus, our observation that BST2 did not inhibit the egress of HCMV could indicate that HCMV developed effective countermeasures. However, while we observed that HCMV counteracted the IFN-dependent induction of BST2, presumably as a result of interfering with JAK/STAT signaling, we observed that HCMV actually induced BST2 upon infection of fibroblasts. Induction of BST2 is likely due to the viral activation of IRF3 via the DNA sensor ZBP-1 which results in induction of IFN and IFN-independent ISG induction [39]. These observations suggest that HCMV does not eliminate BST2 as reported for HIV-1 or KSHV [14], [17]. However, we cannot rule out with certainty that HCMV counteracts the antiviral function of BST2 in a more subtle manner during egress. It is also possible that BST2 is unable to prevent HCMV egress due to differences between cytomegaloviral release and that of viruses susceptible to BST2. In either case, one of the consequences of BST2 upregulation by HCMV seems to be the incorporation of BST2 into the viral envelope. Whether this is a passive incorporation due to the fact that viral envelopes are derived from cellular membranes or due to an active enrichment of BST2 into the viral envelope is currently unknown. Since a previous proteomics study of HCMV virions did not find BST2 in the virion preparations [38] it is possible that BST2 levels vary between virus preparations. Alternatively, the abundance of BST2 was too low to be detected by mass-spectrometry.

In contrast to other enveloped viruses, we observed that BST2 increased HCMV infection. This increase was observed consistently with two viral strains in different cell types using different BST2 construct as well as upon induction of endogenous BST2. Since this increased infection was observed prior to the onset of viral gene expression and even when virus was UV-inactivated, we concluded that BST2 enhanced viral entry. BST2 is highly expressed on monocytes, monocyte-derived macrophages and dendritic cells. Therefore, we propose that BST2 facilitates entry into these cell types, and our data in THP-1 cells and primary monocytes support this. However, the presence of BST2 is not sufficient for HCMV infection of other BST2-expressing cell types such as T cells and B cells, because HCMV replication can be also restricted post-entry (as observed for AD169 in THP-1 cells). Thus, unlike a *bona-fide* receptor, BST2 is not essential for infection because viral infection also occurs in the absence of BST2, particularly in fibroblasts. Instead BST2 seems to act as a co-factor, or co-receptor that facilitates but is not essential for viral entry. In the presence of BST2 a larger percentage of cells seem to be infected rather than an increase of viral replication in individual cells. Therefore, it is conceivable that BST2 increases viral dissemination in vivo by gaining increased access to BST2-expressing cells.

At present, it is unclear how BST2 increases viral entry. However, based on the known function of BST2 as a viral tether preventing viral budding it is conceivable that a similar but reverse mechanism could operate during entry. Interestingly, a report by David Perez-Caballero et al. [40], suggests that the unusual topology of BST2 rather than its primary structure is sufficient for binding and restricting viral release. Thus, replacing the N-terminal transmembrane domain, the extracellular coiled-coil motif and the C-terminal GPI anchor with similar domains from unrelated proteins resulted in a virus-inhibitory protein [40]. Based on these observations, it is hypothesized that BST2 either tethers budding viruses by linking the envelope with the membrane through each of its membrane domains or through dimers that form between virion-associated BST2 and BST2 in the cell membrane. Indeed, electron micrograph analysis revealed BST2 in virion junctures and in the membranes of concatenated virions consistent with either model [40]. Based on these models, we hypothesize that similar tethering mechanisms by BST2 enhance entry of HCMV. Either virion-associated BST2 interacts with BST2 at the cell surface or plasma-membrane-associated BST2 inserts one of its membrane domains into the envelope of the incoming virus, e.g. during membrane fusion. In both cases, BST2 at the cell surface will enhance the virion binding and entry of viruses into the host cells and thereby infection by virus.

Although BST2 is a self interacting protein, BST2 has also been shown to interact with the cellular protein Immunoglobulin-like transcript 7 (ILT-7) at the surface of plasmacytoid dendritic cells [12]. Thus, another potential mechanism for BST2 facilitated entry could be that HCMV encodes a protein that is expressed in the virion membrane and mimics ILT-7 allowing interaction with BST2 on the surface of the target cell. Interestingly, HCMV expresses UL18, an MHC-I like protein that interacts with ILT-2 a protein of the same family as ILT-7 [41].

In summary, our data suggest an unprecedented and unexpected role for BST2 in HCMV entry. This observation is in stark contrast to the well-established role of BST2 in prohibiting egress of a number of unrelated viral families, including at least one herpesvirus [14]. In particular, lentiviruses seem to be highly sensitive to inhibition by BST2 as indicated by the observation that primate lentiviruses generally counteract BST2 of their host species, but are susceptible to inhibition by BST2 from other primate species [23]. In contrast, CMV appears to employ BST2 as an entry factor. Since CMVs co-evolve with their hosts, each CMV strain may adapt to the BST2 of the host species. In vivo, BST2-mediated enhancement of HCMV entry may play a particularly important role in macrophages and dendritic cell that express BST2 upon differentiation in response to infection and inflammation. In addition high levels of BST2 expression have been observed on many cancer cell lines [42] which correlates with reports of high levels of CMV antigen in certain tumors [43]. Thus, BST2 may contribute to CMV tissue tropism. Further studies will delineate these possibilities.

## Materials and Methods

### Ethics statement

Healthy human volunteers who donated blood provided informed written consent before signing research authorization forms that complied with the US Health Insurance Portability and Accountability Act in addition to a medical history questionnaire. These studies were approved by the Institutional Review Board of OHSU.

### Virus and cell culture

Primary human foreskin fibroblast (HFF) cells were obtained from ATCC and cultured in Dulbecco's minimal essential medium (DMEM) supplemented with 10% fetal bovine serum, L-Glutamine and 100 units of penicillin/streptomycin in a humidified incubator with 5% CO_2_ at 37°C. HFF cells used in this study were between passages 8 and 20. HFFs stably transfected with the human telomerase gene (THFs) to extend passage life were obtained from W. Bresnahan (University of Minnesota) [44]. THFs were propagated the same way as HFFs. THP-1 cells were cultured in RPMI with L-Glut, 10% FBS and 100 units of penicillin/streptomycin. HCMV strain AD169 was obtained from the ATCC and Toledo from in house stocks. The viruses were propagated in primary HFFs and purified by centrifugation through a 20% sorbitol cushion for 1 h at 22,000 rpm in a Beckman SW28 rotor. The titers of virus stocks were determined using endpoint serial dilution assays on primary HFFs. For pure virus preparations the supernatant collected from infected cells was cleared of whole cell by spinning at 1,500 rpm, 4°C for 5 min, then the membrane contaminants were removed by two spins at 7,500 rpm, 4°C, 15 min followed by sorbitol cushion purification. This partially cleaned virus was subjected to Nycodenz gradient (50-5% in TNE buffer - 50 mM Tris [pH 7.4], 100 mM NaCl, and 10 mM EDTA). Fractions of the separated gradient were spun at 30,000 rpm at 4°C for 1 h followed by a wash with PBS. The isolated fractions were tested for the presence of viral and cellular proteins, DNA concentration and infectivity. Inactivation of HCMV particles using UV irradiation was performed in a Stratalinker for a length of time sufficient to block expression of protein from the HCMV open reading frame UL123 (IE1) and to induce MHC-I expression as result of IFN activation in the treated cells. Histodenz (Nycodenz) was purchased from Sigma Aldrich.

### Reagents and antibodies

The antibodies pp65, gB (HCMV), CD81 and GAPDH were purchased from Santa Cruz biotechnologies. BST2 rabbit polyclonal antibody was obtained through the AIDS Research and Reference Reagent Program, Division of AIDS, NIAID, NIH from Drs. Klaus Strebel and Amy Andrew (https://www.aidsreagent.org/index.cfm). Anti-HA antibody was obtained from Covance Inc., and the IE1 antibody was from Light Diagnostics. Antibody to pp71 was a gift from Dr Thomas Shenk [45]. Human recombinant IFNβ was obtained from PBL Interferon Source, Piscataway, NJ.

### BST2 expressing stable fibroblasts

Human *bst-2* cDNA was amplified as an NheI/BamHI fragment by a PCR using the *Pfu* enzyme (Stratagene, San Diego, CA) and inserted into the lentiviral vector pCDH-CMV-MCSEF1-Puro (System Biosciences, Mountain View, CA). BST2 and its mutants with a hemagglutinin (HA) tag at position 146 (BST2-HA146) and position 110 (BST2-HA11) were generated by primer-directed mutagenesis using PCR as previously described [14]. Lentiviral supernatants were produced via triple transfection of 293T cells with the pHP-dl-N/A packaging construct, the pHEF-VSVG envelope construct (both constructs were obtained through the AIDS Research and Reference Reagent Program, Division of AIDS, NIAID, NIH, from Lung-Ji Chang), and one of the lentiviral clones described above. Transfections were performed using Effectene (Qiagen, Germantown, MD), with a plasmid ratio of 6∶1∶3 (packaging construct∶envelope construct∶lentiviral clone). After 48 h, the supernatants were collected, and lentiviruses were purified through a 0.8 µm filter. Stable cell lines expressing the previously described constructs BST2HA146 and BST2HA110 [14] were generated by lentiviral transduction and puromycin selection (0.3 µg/ml) in THFs and HFFs. Cells transfected with empty pCDH vector were generated for control.

### PBMC-derived monocyte isolation and activation

Isolation of human peripheral blood monocytes was performed as previously described [33]. Briefly, blood was drawn by venipuncture and centrifuged through a Ficol Histopaque 1077 gradient (Sigma-Aldrich, St. Louis, MO) at 200× g for 30 min at room temperature (RT). Mononuclear cells were collected and washed twice with PBS and 1 mM EDTA to remove platelets at 150× g for 10 min at RT. Monocytes were then layered on top of a 45% and 52.5% iso-osmotic Percoll gradient and centrifuged for 30 min at 400× g at RT yielding monocyte population that was more than >90% pure. Cells were washed twice with saline at 150× g for 10 min at RT to remove residual Percoll and suspended in RPMI 1640 (Cellgro) supplemented with 10% human serum (Sigma-Aldrich).

### Virus infection

To monitor virus entry, HCMV was added to cells at an MOI of 3 unless otherwise noted and rocked in an incubator with CO_2_ at 37°C for 1 h. Then the cells were washed with citric acid buffer (pH = 3) followed by three cold PBS washes and lysed in Laemmli buffer with 10% β-mercaptoethanol. The lysates were analyzed for the expression of tegument proteins pp65 or pp71 using previously described antibodies. For analysis of immediate early gene expression infected cells were harvested 8hpi and lysates subjected to immunoblot. For the time course analysis with AD169-GFP virus, the cells were treated with the virus at an MOI of 2 and analyzed for GFP positive cells over time. SiRNA treatment was performed 2 days prior to cell infection with AD169-GFP at MOI of 1 and analyzed for GFP positive cells 24hpi. THP-1 cells and 293-M cells were infected with HCMV-AD169 at an MOI of 3 and harvested at early times (3 h) to detect tegument proteins or at a later times (24 h) for analysis of IE1 protein expression. PBMC derived monocytes were infected with HCMV Toledo at an MOI of 5. Relative genome copies were measured in THP-1 cells activated using PMA and infected with HCMV-TR strain after SiRNA treatments.

### SiRNA transfections

SiRNA against BST2, GAPDH and SiGlo were obtained from Dharmacon Inc., Lafayette, CO (Smart pool) and negative control siRNAs (-ve SiRNA) from Qiagen. HFF cells were treated with Lipofectamine 2000 (3 µL/mL; Invitrogen) and SiRNAs at a concentration between 10–40 nM twice 72 h apart. THP-1 cells were treated with Hyperfect (10 µL/mL; Invitrogen) and SiRNA at 40 nM after 24 h of treatment with PMA (1 µg/mL). PBMC-derived monocytes were treated with RNAi Max (10 µL/mL; Invitrogen) after adhering to plastic with 50 nM of the SiRNAs for 48 h and infected with HCMV for 4 h or 24 h. The cells were probed for pp65 or IE1 proteins.

### Immunofluorescence and flow cytometry

THP-1 cells were treated with 1 µg/ml of PMA for 24 h followed by transfection with SiBST2. After three days, the cells were infected with HCMV and washed with PBS after 24 hours followed by fixing with 2% paraformaldehyde and incubating with antibodies for IE1 and BST2. Slides were fixed a second time in 2% paraformaldehyde after the final antibody treatment and washed three times with PBS. Coverslips were then mounted on slides and covered with Vectashield H-1200 þ DAPI (Vector Laboratories).

For flow cytometry, cells were removed from tissue culture dishes with 0.05% trypsin-EDTA (Invitrogen), washed with ice-cold PBS, and fixed with 3.7% formaldehyde solution. For intracellular staining, cells were permeabilized using perm wash solution (1% saponin, 10% NaN_3_, 10% fetal calf serum [FCS], in PBS). For surface staining the cells were directly incubated with appropriate primary antibody for 60 min at 4–8°C. The cells were washed with ice-cold PBS and either resuspended in ice-cold PBS or incubated with PE-conjugated anti-mouse secondary antibody (Dako, http://www.dako.com) and washed twice again before analyzed using FACSCalibur flow cytometer (BD Biosciences).

### Quantitative PCR

Total mRNA from cells was isolated and purified using RNeasy (Qiagen). Specific primers for BST2 and β-actin were designed using Primer 3 software (BST2 primers CCGTCCTGCTCGGCTTT [forward] and CCGCTCAGAACTGATGAGATCA [reverse]; β-actin primers - TCACCCACACTGTGCCCATCTACGA [forward] and GCGGAACCGCTCATTGCCAATGG [reverse]). Transcript levels were determined by quantitative real-time reverse transcriptase PCR (qPCR), using SYBR green dye incorporation (AB Applied Biosystems, Warrington, United Kingdom) and AmpliTaq Gold DNA polymerase in an ABI Prism 7900HT sequence detection system (AB Applied Biosystems, Warrington, United Kingdom). The comparative threshold cycle method was used to derive the change in gene expression between different treatments, using β-actin as an internal standard.

To quantify the number of viral genomes in DNA isolated from infected cells we used a Taqman primer probe set that amplifies the UL81/82 region of the HCMV genome (Fwd - GAGGTAGGTCGTAGTGCGGC ; Rev- GCTCTCACGCTCGTCATCC ; probe: TGCTGCACGCTCAC with VIC reporter). 50 ng of total cellular DNA isolated from infected cells after citric acid wash was used as template. A standard curve was determined using DNA isolated from the input virus. The DNA was amplified on a ABI step one plus and the data were analyzed using step one software v2.1.

### Western blot analysis and quantification

The samples were prepared in Laemmli buffer with 10% β-mercaptoethanol and separated using 10% SDS-PAGE. The proteins were transferred to polyvinylidene difluoride membrane (Waters Ltd., Milford, MA) and probed with primary antibodies for 1 h at room temperature, followed by horseradish peroxidase-conjugated secondary antibody (Santa Cruz Antibody Solutions). Membranes were washed in 0.1% Tween 20 in PBS. The proteins were detected using a SuperSignal West Femto chemiluminescence kit (Thermo Scientific, Rockford, IL). The bands were quantified using Image J software from NCBI (http://rsbweb.nih.gov/ij/). The images were converted into the binary mode and ratios were derived by comparing viral protein band to the host control protein band (GAPDH) and represented as graphs next to the blots. For detecting BST2 in virion sample, it was deglycosylated using PNGaseF (New England biolabs inc.,).

### Electron microscopy: Immunogold labeling of gradient purified HCMV virions

Approximately 10^8^ particles were applied to carbon-coated gold 300-mesh grids. The virions were fixed in 4% paraformaldehyde in PBS and grids were washed three times in PBS, blocked in 5% BSA, 2% normal goat serum in PBS (pH 7.4) followed by incubation with antibodies to BST2 or HCMV gB for one hour. Then the grids were washed and incubated with secondary 20-nm gold conjugated anti-mouse antibodies (Ted Pella Inc., Redding, CA) diluted 1∶20 in blocking solution. The samples were stained with ammonium molybdate and examined on a Philips EM 300 electron microscope. For statistical analysis gold positive virus particles were counted and presented as percentage of total virus particles.

### Statistical analysis

The bar graphs for viral entry assay represent mean ± SD from 3 to 5 replicates for each experiment. Significance was assessed by analysis of variance (ANOVA) with secondary Fishers least significant difference (FLSD) and Mann Whitney; P values<0.05 considered significant.
